# *APOE* Genotype Differentially Modulates Effects of Anti-Aβ, Passive Immunization in APP Transgenic Mice

**DOI:** 10.1186/s13024-017-0156-1

**Published:** 2017-01-31

**Authors:** Joanna E Pankiewicz, Jairo Baquero-Buitrago, Sandrine Sanchez, Jennifer Lopez-Contreras, Jungsu Kim, Patrick M. Sullivan, David M. Holtzman, Martin J. Sadowski

**Affiliations:** 1 Department of Neurology, New York University School of Medicine, New York, NY 10016 USA; 2 Department of Biochemistry and Molecular Pharmacology, New York University School of Medicine, New York, NY 10016 USA; 3 Department of Psychiatry, New York University School of Medicine, New York, NY 10016 USA; 4 Department of Neurology, Washington University School of Medicine, St. Louis, MO 63110 USA; 5 Knight Alzheimer’s Disease Research Center, Washington University School of Medicine, St. Louis, MO 63110 USA; 6 Hope Center for Neurological Disorders, Washington University School of Medicine, St. Louis, MO 63110 USA; 7 Department of Neuroscience, Mayo Clinic College of Medicine, Jacksonville, FL 32224 USA; 8 Department of Medicine (Geriatrics), Duke University School of Medicine, Durham, NC 27710 USA; 9 Durham VA Medical Center’s Geriatric Research, Education, and Clinical Center, Durham, NC 27710 USA

**Keywords:** Alzheimer’s disease, Apolipoprotein E, β-amyloid, Brain hemorrhages, Microglia, Neurodegeneration, Therapy, Vasculopathy

## Abstract

**Background:**

*APOE* genotype is the foremost genetic factor modulating β-amyloid (Aβ) deposition and risk of sporadic Alzheimer’s disease (AD). Here we investigated how *APOE* genotype influences response to anti-Aβ immunotherapy.

**Methods:**

APP_SW_/PS1_dE9_ (APP) transgenic mice with targeted replacement of the murine *Apoe* gene for human *APOE* alleles received 10D5 anti-Aβ or TY11-15 isotype control antibodies between the ages of 12 and 15 months.

**Results:**

Anti-Aβ immunization decreased both the load of fibrillar plaques and the load of Aβ immunopositive plaques in mice of all *APOE* backgrounds. Although the relative reduction in parenchymal Aβ plaque load was comparable across all *APOE* genotypes, APP/ε4 mice showed the greatest reduction in the absolute Aβ plaque load values, given their highest baseline. The immunization stimulated phagocytic activation of microglia, which magnitude adjusted for the post-treatment plaque load was the greatest in APP/ε4 mice implying association between the ε4 allele and impaired Aβ phagocytosis. Perivascular hemosiderin deposits reflecting ensued microhemorrhages were associated with vascular Aβ (VAβ) and ubiquitously present in control mice of all *APOE* genotypes, although in APP/ε3 mice their incidence was the lowest. Anti-Aβ immunization significantly reduced VAβ burden but increased the number of hemosiderin deposits across all *APOE* genotypes with the strongest and the weakest effect in APP/ε2 and APP/ε3 mice, respectively.

**Conclusions:**

Our studies indicate that *APOE* genotype differentially modulates microglia activation and Aβ plaque load reduction during anti-Aβ immunotherapy. The *APOE* ε3 allele shows strong protective effect against immunotherapy associated microhemorrhages; while, conversely, the *APOE* ε2 allele increases risk thereof.

**Electronic supplementary material:**

The online version of this article (doi:10.1186/s13024-017-0156-1) contains supplementary material, which is available to authorized users.

## Background

Accumulation of β-amyloid (Aβ) in the brain is a culprit early in Alzheimer’s disease (AD) pathogenesis, and triggers down-stream neurodegenerative cascades including inflammatory responses, intraneuronal neurofibrillary pathology, and synaptic and neuronal loss (reviewed in [1–3]). Susceptibility to sporadic AD is foremost modulated by *APOE* genotype, which also influences the load of Aβ parenchymal plaques and vascular Aβ (VAβ) deposits. A single copy of the *APOE* ε4 allele endows a ~3 fold increase in AD risk, and 2 *APOE* ε4 copies result in a ~15 fold risk increase, while an *APOE* ε2 allele halves AD risk relative to 2 copies of ε3 (reviewed in [4]). Autopsy series and more recently positron emission tomography (PET) imaging studies of fibrillar Aβ plaque load in AD patients have shown ε4> > ε3 > ε2 allele gradation effect on Aβ deposition [1–3]. To counteract down-stream neurodegenerative effects triggered by Aβ accumulation, development of anti-Aβ therapeutic strategies including anti-Aβ immunotherapy have been proposed and pursued. Several anti-Aβ monoclonal antibodies (mAbs) have been tested in clinical trials in AD patients and were found to significantly reduce load of fibrillar Aβ as demonstrated using PET Aβ imaging [5–8]. Whether *APOE* genotype also differentially modulates degree of Aβ plaque load reduction in response to anti-Aβ passive immunization remains unknown due to limited clinical data and because preclinical testing of anti-Aβ mAbs has been exclusively conducted in AD transgenic (Tg) mice models expressing wild type, murine apoE [9–12]. The main adverse effects associated with administration of certain anti-Aβ mAbs during clinical trials were amyloid related imaging abnormalities (ARIA) identified on magnetic resonance imaging (MRI) scans. These included vasogenic edema (ARIA-E) and cerebral microhemorrhages (ARIA-H) that in about 20% of cases are associated with clinical symptoms and signs [13, 14]. Frequency of ARIA events and in particular ARIA-E was significantly higher among *APOE* ε4 allele carriers compared to non-carriers, making the *APOE* ε4 allele a risk factor for vascular complications of anti-Aβ immunotherapy [5, 8, 14, 15]. In view of these considerations, we sought to re-examine the effects of passive immunization in APP_SWE_/PS1_dE9_ Tg mice with targeted replacement of the murine *Apoe* gene for various human *APOE* alleles, which expression remains controlled by the native murine *Apoe* promoter [16, 17]. These Tg mouse lines, hereafter designated as APP/ε2, APP/ε3, and APP/ε4, reflect the differential effect of *APOE* alleles on the load of Aβ parenchymal plaques and VAβ known from AD patients [18]. The vaccination experiment was started in 12 months. old mice with advanced load of Aβ deposits [19, 20], to emulate the stage of human disease in regard to Aβ deposition in which anti-Aβ immunotherapy is currently occurring. We used mAb 10D5 direct against Aβ_3–7_ epitope [10], which is known to penetrate the blood–brain-barrier (BBB) and directly binds to deposited Aβ triggering microglial cells to clear Aβ plaques through Fc receptor-mediated phagocytosis [9]. Our study in *APOE* humanized APP_SWE_/PS1_dE9_ mice provides evidence for differential effect of *APOE* alleles on response to anti-Aβ immunotherapy and occurrence of vascular complications associated with thereof.

## Methods

All reagents and antibodies, unless stated otherwise, were purchased from Sigma-Aldrich (St. Louis, MO).

### Animals and antibody treatment

All mouse care and experimental procedures were approved by Institutional Animal Care and Use Committees of the New York University School of Medicine and the Washington University School of Medicine. Generation of APP/ε2, APP/ε3, and APP/ε4 mice by cross-breeding of *APOE* ε2, *APOE* ε3, or *APOE* ε4 targeted replacement mice with APP_SW_/PS1_dE9_ mice and detailed genotyping procedures have been previously described [19, 20]. This study was performed using non-breeder mice of both sexes with each sex contributing approximately half to the total animal number in each experimental group. Animals separated by sex were aged in a barrier facility with a 12/12 h. light/dark cycle and ad libitum food and water access. The antibody treatment in animals of all *APOE* backgrounds was commenced at the age of 12 months and was carried out continuously for three months till the animals were killed at the age of 15 months. Anti-Aβ 10D5 mAb or TY11-15 an isotype control IgG2a antibody [9, 10, 21] were administered once a week (10 mg/kg) through intraperitoneal injections. The dose of 10D5 mAb used in this study was previously shown to be effective in removing Aβ plaques in PDAPP AD Tg mice expressing wild type murine apoE [9, 10, 22]. Prior to each injection mice were weighted and the accurate dose of administered antibodies was quantified. Antibodies were injected diluted in 0.25 ml of sterile phosphate buffered saline (PBS), pH 7.4 using a 29 gauge needle. Both 10D5 mAb and TY11-15 control IgG were produced, purified and provided by Janssen Alzheimer Immunotherapy. In addition to 10D5 mAb treatment and TY11-15 control groups, for each *APOE* genotype we analyzed a group of age-matched mice receiving no treatment or sham injections. During the treatment all animals were closely monitored for any signs of toxicity by the veterinary staff. 10D5 mAb and TY11-15 control IgG2a injections were well tolerated by the animals, which showed no abnormalities in the following monitored parameters: body weight, physical appearance, occurrence of unprovoked behavior, and blunted or exaggerated responses to external stimuli. At the age of 15 months all animals were killed with an overdose of sodium pentobarbital (150 mg/kg) and transcardially perfused with heparinized (1,000 units/L) 0.01 M PBS at pH 7.4 and temperature 37^O^ C.

### Histological processing and immunohistochemistry

Brains were extracted from skulls and fixed by immersion in 4% paraformaldehyde in 0.1 M phosphate buffer at pH 7.4 for 72 h. and then dehydrated in a mixture of 20% Dimethyl sulfoxide and 20% glycerol in 0.01 PBS at pH 7.4 and temperature 4^O^ C. Brains were cut serially along the entire rostro-caudal axis using a freezing microtome (Leica Microsystems, Wetzlar, Germany), into coronal, 40-μm-thick sections, which were alternately collected into 10 series. Randomly selected series of sections were used for the following histological and immunohistochemical procedures: Thioflavin-S (Th-S) stain to visualize fibrillar component of Aβ parenchymal plaques and cerebral amyloid angiopathy (CAA); anti-Aβ immunohistochemistry using HJ3.4 mAb (1:250) [19, 23]; Perls’ Prussian blue stain to detect perivascular ferric iron deposition [24–26]; and a combination of anti-Iba1 immunostaining (anti-Iba1 rabbit polyclonal antibody 1:1,000; Wako Chemicals USA Inc., Richmond VA) or anti-CD68 immunostaining (anti-CD68 rabbit polyclonal antibody 1:250; Abcam Inc., Cambridge, MA) and Th-S to characterize microglia response in association with fibrillar Aβ parenchymal plaques. The intensity of HJ3.4 immunostaining was enhanced by formic acid (FA) pretreatment and the immunohistochemistry protocol was concluded using M.O.M. 3,3'-diaminobenzidine (DAB) kit (Vector Laboratories; Burlingame, CA) as per manufacturer manual. The anti-CD68 and anti-Iba1 immunostaining protocol was concluded using mouse anti-rabbit IgG biotinylated antibody (1:500) followed by streptavidin conjugated Cy3 fluorochrome (1:200), as previously described [19]. Additional series from selected brains were double-stained with Perls’ Prussian blue stain and HJ3.4 anti-Aβ mAb or Th-S stain to visualize association of VAβ with hemosiderin deposits. One series of sections from five randomly selected APP/ε4 mice treated with 10D mAb and TY11-15 IgG controls were immunostained with 4G8 mAb (1:2,000) (Covance; Princeton, NJ) directed against Aβ residues 17–24 [20, 27]. In this case, sections were also FA pretreated to enhance Aβ immunodetection and the staining procedure was concluded using M.O.M. DAB kit. This was done to verify that 10D5 mAb does not obscure binding of HJ3.4 mAb to deposited Aβ since both mAbs are directed against the N-terminus of Aβ while 4G8 mAb binds a central Aβ epitope.

### Analysis of fibrillar parenchymal plaque load and cerebral amyloid angiopathy (CAA) load

**Fig. 1:**
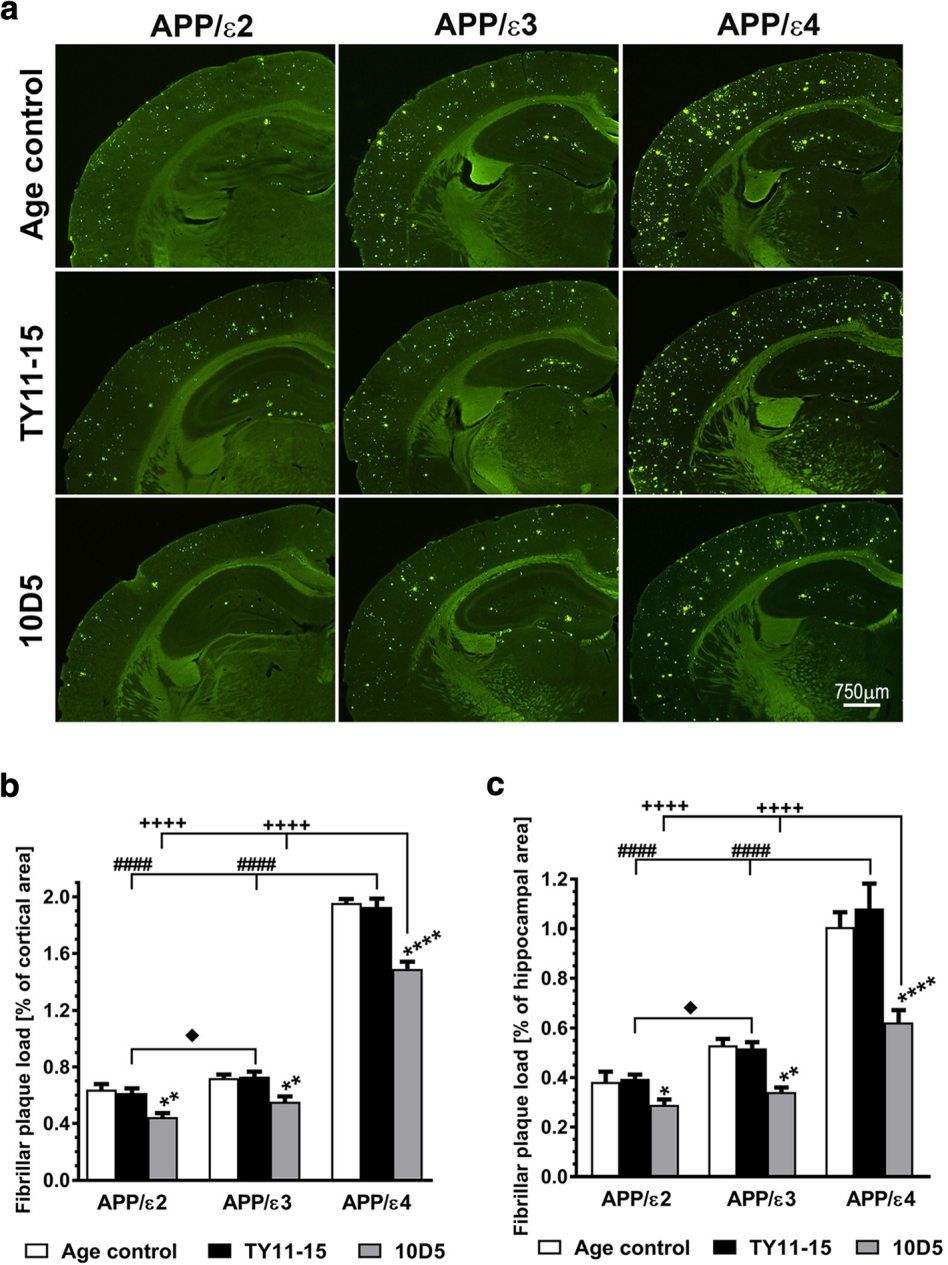
Analysis of the fibrillar plaque load. *APOE* genotype differentially modulates fibrillar plaque load and its reduction in response to 10D5 mAb treatment. **a** Representative microphotographs of Thioflavin-S (Th-S) stained coronal brain sections at the level of the rostral hippocampus from untreated age-matched (Age control) mice, and mice receiving an isotype control IgG2a antibody TY11-15 or 10D5 anti-Aβ mAb. Unbiased quantification of Th-S positive, fibrillar Aβ plaque load in the brain cortex (**b**) and in the hippocampus (**c**). Values shown in (**b**) and (**c**) represent mean ± SEM from 7 to 13 animals in TY11-15 and 10D5 mAb treated groups and 5 to 11 mice in Age control groups per *APOE* genotype. (**b**), and (**c**) *p* < 0.0001 (one-way analysis of variance); *p < 0.05, **p < 0.01, and ****p < 0.0001, TY11-15 control vs. 10D5 mAb treatment for matching *APOE* genotypes (Sidak’s post hoc test). Differences between Age control and TY11-15 groups were non-significant for all matching *APOE* genotypes (Sidak’s post hoc test); not shown on the graph. ^####^ *p* < 0.0001, TY11-15 APP/ε4 control vs. TY11-15 APP/ε2 or APP/ε3 controls (Sidak’s post hoc test). ^♦^ *p* < 0.05, TY11-15 APP/ε2 control vs. TY11-15 APP/ε3 control (Sidak’s post hoc test). ^++++^ *p* < 0.0001, 10D5 mAb treated APP/ε4 mice vs. 10D5 mAb treated APP/ε2 or APP/ε3 mice (Sidak’s post hoc test). Differences between 10D5 mAb treated APP/ε2 and APP/ε3 mice were non-significant (Sidak’s post hoc test); not shown on the graph. Scale bars 750 μm (**a**)

These metrics were determined using an unbiased, whole-section quantification approach. The entire cross-sectional profile of the right hemisphere stained with Th-S, was photographed under × 4 objective magnification using a high-sensitivity, cooled, monochrome DS-Qi1Mc camera attached to 80i Nikon fluorescent microscope (Nikon Corp. Tokyo, Japan). Adjacent frames were combined into a single image using the photo-stitching function of the NIS Elements imaging software (Nikon Corp. Tokyo, Japan) and analyzed with the help of NIH Image J v 1.47 image processing software package (Bethesda, MD). The cross-sectional profiles of the brain cortex or the hippocampus was manually outlined on the hemispheric images and the load of all Th-S positive Aβ deposits (defined as the percentage of a brain structure profile occupied by Th-S positive lesions) was automatically thresholded and filtered according to the preset algorithm to discriminate nonspecific staining. Then Th-S positive vascular deposits constituting CAA were manually eliminated from digitized images and the thresholding procedure was repeated to quantify the load of fibrillar parenchymal Aβ plaques only. The CAA load was then calculated as the difference between the load of all Th-S positive Aβ deposits and the load of Th-S positive parenchymal Aβ plaques for a given brain section. The brain cortex was analyzed on three coronal cross-sectional profiles per brain, which were taken from levels of the anterior commissure, the rostral portion of the hippocampus (Fig 1a), and the mammillary bodies and correspond to the following stereotactic coordinates: Bregma 0 mm, Bregma −1.25 mm, and Bregma −2.8 mm, respectively. The hippocampus was analyzed on serial coronal cross-sectional profiles through the dorsal hippocampus.

### Quantification of immunopositive Aβ parenchymal plaque load

The load of Aβ plaques immunostained with HJ3.4 or 4G8 anti-Aβ mAbs in the brain cortex was analyzed on three coronal cross-sectional profiles per brain corresponding to the aforementioned anatomical levels. The load of immunopositive Aβ plaques in the hippocampus was analyzed on serial coronal cross-sectional profiles through the dorsal hippocampus. Serial microphotographs covering the entire cross-sectional profile of the brain cortex or the hippocampus were taken under ×20 magnification using DS-Fi1 color camera attached to 80i Nikon microscope. Captured photographs were combined into a single image using the photo-stitching function of the NIS Elements Imaging Software, and automatically thresholded and filtered by size according to the preset algorithm to discriminate nonspecific staining and Aβ positive capillaries using NIH Image J v 1.47 (Bethesda, MD). The load of immunopositive parenchymal Aβ plaques per brain was calculated as the average of load values obtained from all three analyzed brain sections.

### Assessment of cerebral capillary Aβ (CCA)

Prevalence of HJ3.4-immunoreactive capillaries was analyzed on three coronal cross-sectional profiles through the brain cortex at the aforementioned anatomical levels and on three to four subsequent coronal cross-sectional profiles through the thalamus. Aβ immunopositive capillaries (defined as vessels with diameter < 8 μm [28, 29]) were manually counted at 40× objective magnification and their number was expressed as n/mm^2^.

### Microglia analysis

The analysis was performed on sections immunostained against Iba1 or CD68 antigen and counterstained with Th-S. Microphotographs of the brain cortex were taken at × 20 objective magnification under Tetramethylrhodamine (for Iba1/Cy3 and CD68/Cy3) and Fluorescein isothiocyanate (for Th-S) channels of 80i Nikon fluorescent microscope (Nikon Corp. Tokyo, Japan) using DS-Qi1Mc monochrome camera (Nikon Corp. Tokyo, Japan). The load of Iba1 and CD68 positive cells (defined as the percentage of a microphotograph area occupied by anti-Iba1 or anti-CD68 positive immunostaining) was automatically thresholded and filtered according to the preset algorithm to discriminate nonspecific staining using NIH Image J v 1.47 (Bethesda, MD). To control for variable Aβ parenchymal plaque load across *APOE* genotypes the Iba1 load and the CD68 load was divided by the load of Th-S positive Aβ parenchymal plaques determined on the same microphotograph as described above.

### Analysis of perivascular hemosiderin deposits

A complete series of brain coronal cross-sections evenly spaced 400 μm apart from each animal was analyzed. Such series typically includes 13–15 sections, which were stained with Perls’ Prussian blue method without counterstain to maximize sensitivity of detection. Manual count of perivascular hemosiderin deposits in all brain structures (Additional file 2: Figure S2 A-C) was performed under × 20 objective magnifications. Deposits ≥ 15 μm in diameter were classified as large.

### Measurement of the serum cholesterol level

At the conclusion of the experiment blood samples were collected and centrifuged to separate serum. The total serum cholesterol concentration was measured using a standard enzymatic assay based on the Cholesterol E kit (Wako Diagnostics, Richmond, VA) as previously described [19, 24].

### Statistical analysis

Distribution of data within all data sets was analyzed with Kolmogorov-Smirnov and Shapiro-Wilk tests. Multiple data sets conforming to the normal distribution were first compared for statistical differences using one-way analysis of variance, which was then followed by Sidak's post hoc test for comparison between selected data sets. Multiple data sets, which did not conform to the normal distribution, were compared using Kruskal-Wallis one-way analysis of variance by ranks followed by Dunn’s test for post hoc comparison between selected data sets. All statistical analyses were performed using GraphPad Prism v 6.05 (GraphPad Software, Inc.). All data were reported as the mean and the standard error of the mean (SEM) per *APOE* genotype and treatment arm for each outcome measure.

## Results

### *APOE* genotype modulates reduction in parenchymal plaque load by Aβ immunotherapy

**Fig. 2:**
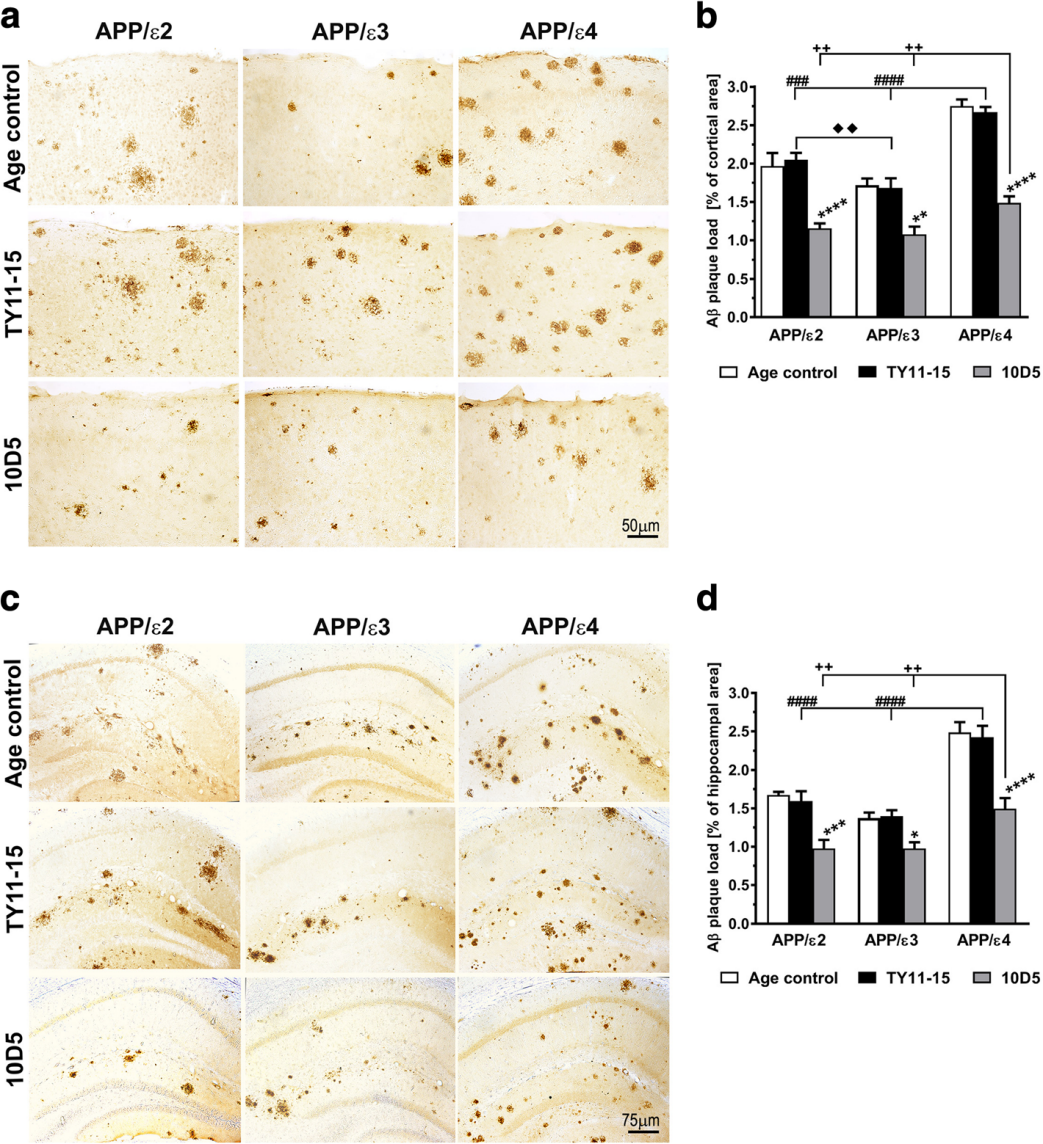
Analysis of the parenchymal Aβ plaque load. Differential effects of *APOE* genotype on Aβ plaque load and its reduction effected by 10D5 mAb treatment. Representative microphotographs of coronal brain sections through the somatosensory cortex (**a**) and the dorsal hippocampus (**c**) from untreated age-matched (Age control) mice, and mice receiving an isotype control IgG2a antibody TY11-15 or 10D5 anti-Aβ mAb. Aβ plaques were immunostained with an antibody against the N-terminus of Aβ following formic acid treatment of the sections. Unbiased analysis of the parenchymal Aβ plaque load in the brain cortex (**b**), and in the hippocampus (**d**) revealed by anti-Aβ immunostaining. Values shown in (**b**) and (**d**) represent mean ± SEM from 8 to 12 animals in TY11-15 and 10D5 mAb treated groups and 5 to 10 mice in Age control groups per *APOE* genotype. (**b**) and (**d**) *p* < 0.0001 (one-way analysis of variance); *p < 0.05, **p < 0.01, and ****p < 0.0001, TY11-15 control vs. 10D5 mAb treatment for matching *APOE* genotypes (Sidak’s post hoc test). Differences between Age control and TY11-15 groups were non-significant for all matching *APOE* genotypes (Sidak’s post hoc test); not shown on the graph. ^###^ *p* < 0.001 and ^####^ *p* < 0.0001, TY11-15 APP/ε4 control vs. TY11-15 APP/ε2 or APP/ε3 controls (Sidak’s post hoc test). ^♦♦^ *p* < 0.01, TY11-15 APP/ε2 control vs. TY11-15 APP/ε3 control (Sidak’s post hoc test). ^++^ *p* < 0.01, 10D5 mAb treated APP/ε4 mice vs. 10D5 mAb treated APP/ε2 or APP/ε3 mice (Sidak’s post hoc test). Differences between 10D5 mAb treated APP/ε2 and APP/ε3 mice were non-significant (Sidak’s post hoc test); not shown on the graph. Scale bars 50 μm (*A*) and 75 μm (**c**)

*APOE* targeted replacement APP_SW_/PS1_dE9_ mice faithfully reflect the differential effect of *APOE* alleles on Aβ brain deposition. At the age of 15 months, the load of Th-S positive, fibrillar plaques in the brain cortex of TY11-15 control APP/ε4 mice was 3.1 and 2.6 fold higher than that in TY11-15 control APP/ε2 and APP/ε3 mice, respectively, while in the hippocampus it was 2.7 and 2.1 fold higher, respectively (*p* < 0.0001) (Fig. 1a-c). The load of immunopositive Aβ plaques in the brain cortex of TY11-15 control APP/ε4 mice was 1.3 and 1.6 fold higher, than that in TY11-15 control APP/ε2 and APP/ε3 mice, respectively, while in the hippocampus it was 1.5 and 1.7 fold higher, respectively (*p* < 0.001) (Fig. 2a-d). The plaque load in the brain cortex for matching *APOE* genotypes was higher than that in the hippocampus and the fold difference ranged from 1.4 (APP/ε3) to 1.8 (APP/ε4) for fibrillar plaque load (*p* < 0.01) and from 1.1 (APP/ε4) to 1.3 (APP/ε2) for immunopositive Aβ plaque load (*p* < 0.05 for APP/ε2 only). As we found no statistically significant differences in the load of fibrillar and immunopositive Aβ plaques between male and female mice of the same *APOE* genotype, thus animals of both sexes were combined for all analyses.

10D5 mAb immunization decreased both fibrillar and immmunopositive Aβ parenchymal plaque loads. The degree of reduction relative to TY11-15 controls was comparable across all three *APOE* genotypes. Thus, the fibrillar plaque load in the brain cortex in 10D5 mAb treated APP/ε2, APP/ε3, APP/ε4 mice was reduced by 27.7% (*p* < 0.01), 24.4% (*p* < 0.01), and 22.7% (*p* < 0.0001) of the TY11-15 control mean value for matching *APOE* genotype (Fig. 1a, b), respectively; while the load of Aβ immunopositive plaques was reduced by 43.7% (*p* < 0.0001), 36.0% (*p* < 0.01), and 44.3% (*p* < 0.0001) (Fig. 2a, b), respectively. Similarly, the load of fibrillar plaques in the hippocampus of 10D5 mAb treated APP/ε2, APP/ε3, APP/ε4 mice was decreased by 26.7% (*p* < 0.05), 34.0% (*p* < 0.01), and 42.5% (*p* < 0.0001) of the TY11-15 control mean value for matching *APOE* genotype (Fig. 1a, c), respectively; while the load of Aβ immunopositive plaques was lowered by 38.7% (*p* < 0.001), 30.1% (*p* < 0.05), and 38.3% (*p* < 0.0001) (Fig. 2c, d), respectively. However, given significantly higher plaque load baseline in APP/ε4 mice, the absolute plaque load reduction effected by same dose of 10D5 mAb was incomparably higher in APP/ε4 mice than in other genotypes. Thus, the fibrillar plaque load in the brain cortex was reduced from 1.93 ± 0.05% of the cortical cross-sectional area in TY11-15 APP/ε4 controls to 1.49 ± 0.06% in 10D5 mAb treated APP/ε4 mice (absolute reduction = 0.44%), while the load of immunopositive Aβ plaques was reduced from 2.67 ± 0.07% to 1.49 ± 0.09% between TY11-15 and 10D5 mAb groups, respectively (absolute reduction = 1.18%). For comparison in APP/ε2 and APP/ε3 mice the absolute reduction in the fibrillar plaque load in the brain cortex was 0.17% and 0.18%, respectively; while the absolute reduction in the load of Aβ immunopositive plaques was 0.89% and 0.61%, respectively. Analogously, the fibrillar plaque load in the hippocampus of APP/ε4 mice was reduced from 1.08 ± 0.10% of the hippocampal cross-sectional area in TY11-15 controls to 0.62 ± 0.05% in 10D5 mAb treated mice (absolute reduction = 0.46%), while the load of immunopositive Aβ plaques was reduced from 2.43 ± 0.15% to 1.50 ± 0.14% between TY11-15 and 10D5 mAb groups, respectively (absolute reduction = 0.93%). In APP/ε2 and APP/ε3 mice the absolute reduction in fibrillar plaque load in the hippocampus was 0.11% and 0.18%, respectively; while the absolute reduction in the load of Aβ immunopositive plaques was 0.62% and 0.42%, respectively. Despite greater treatment effect seen in APP/ε4 mice, post-treatment load of both fibrillar and immunopositive Aβ parenchymal plaques in APP/ε4 mice remained significantly higher than these in 10D5 mAb treated APP/ε2 and APP/ε3 mice (Fig. 1b, c and Fig. 2b, d).

We found no statistically significant differences between values of fibrillar plaque loads in the brain cortex and in the hippocampus between TY11-15 isotype control IgG2a antibody treated mice and untreated age-matched mice for corresponding *APOE* genotypes (Fig. 1a-c). Likewise the loads of immunopositive Aβ plaques in TY11-15 control and untreated age-matched mice for corresponding *APOE* genotypes were similar (Fig. 2a-e).

Since 10D5 mAb used for immunotherapy and HJ3.4 mAb used for immunohistochemistry are both directed against the N-terminus of Aβ we immunostained brain section from TY11-15 control and 10D5 mAb treated APP/ε4 mice with 4G8 mAb directed against mid-portion of Aβ [27] and compared Aβ plaque load on immunostaining with both antibodies. This analysis was done to eliminate unlikely possibility that 10D5 mAb hinders the HJ3.4 epitope resulting in reduced ability to detect plaques. Average values of parenchymal Aβ plaque loads revealed by 4G8 mAb immunostaining were 2.57% ± 0.10% and 1.49% ± 0.09 in TY11-15 control and 10D5 mAb treated APP/ε4 groups; respectively; and they were not significantly different from values obtained with HJ3.4 mAb for the same animal groups (Additional file 1: Fig. S1 A, B).

### 10D5 mAb exerts enhanced effector response on microglia in the setting of the APOE ε4 allele

**Fig. 3:**
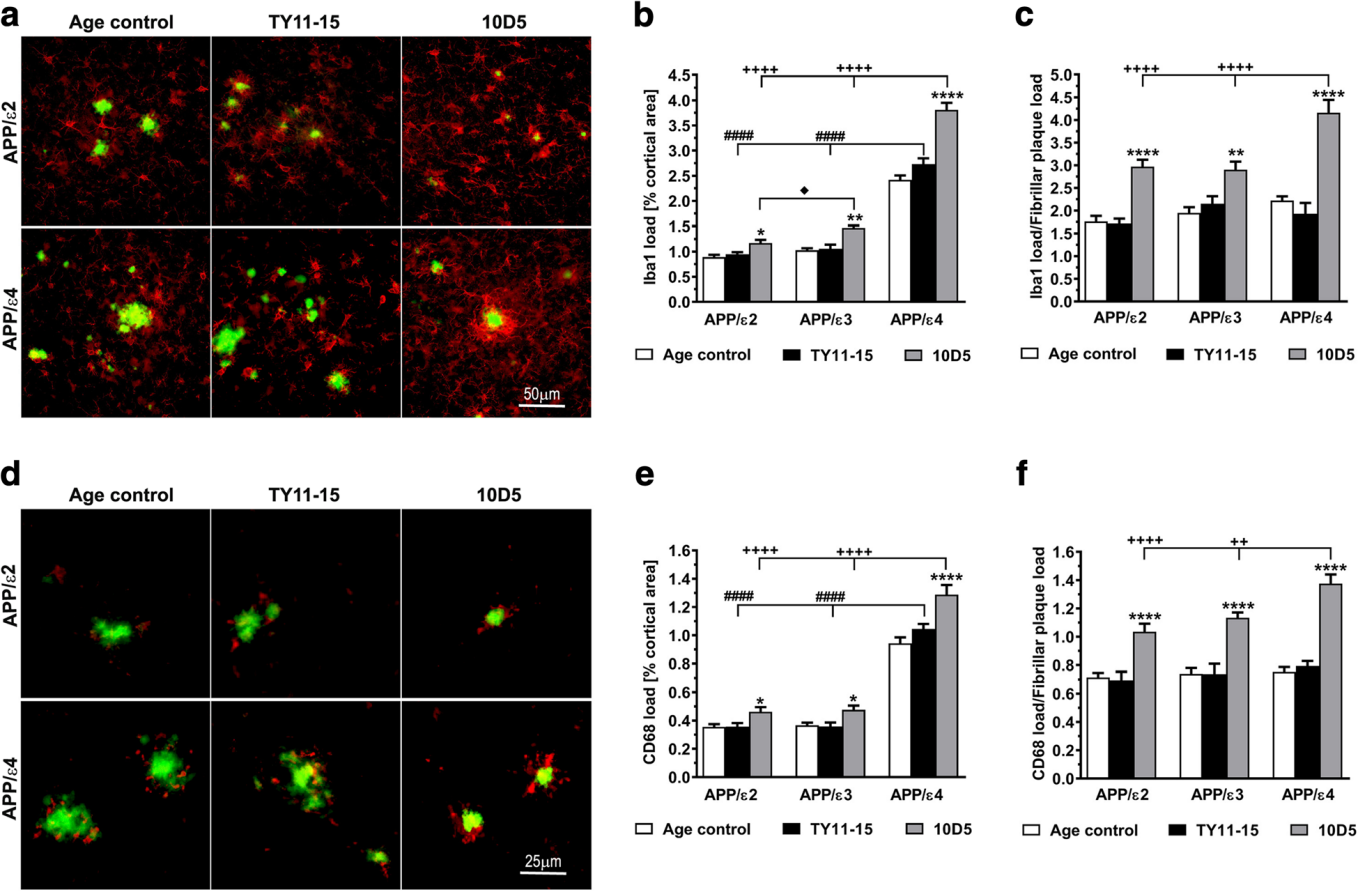
Characterization of microglia response. The *APOE* ε4 allele is associated with enhanced microglia activation resulting from 10D5 mAb immunization compared to other *APOE* genotypes. **a** Representative microphotographs of Iba1 positive microglia in the brain cortex from untreated Age control APP/ε2 and APP/ε4 mice, and APP/ε2 and APP/ε4 mice receiving TY11-15 an IgG2a isotype control antibody or 10D5 anti-Aβ mAb. Anti-Iba1 immunostaining is visualized by red color while green color reflects counterstaining of fibrillar Aβ parenchymal plaques with Th-S. **b** Unbiased quantification of Iba1 positive cell load in the neocortex and (**c**) Iba1 load normalized to Th-S positive fibrillar plaque load across *APOE* genotypes and treatment arms. **d** Representative microphotographs of CD68 positive microglia associated with Aβ parenchymal plaques from Age control, TY11-15 or 10D5 treated APP/ε2 and APP/ε4 mice. Anti-CD68 immunostaining and Th-S staining of fibrillar Aβ plaques are visualized by red and green colors, respectively. **e** Unbiased quantification of CD68 positive cell load in the neocortex and (**f**) CD68 positive cell load normalized to Th-S positive parenchymal plaque load across *APOE* genotypes and treatment arms. Values shown in (**b**), (**c**), (**e**), and (**f**) represent mean ± SEM from n = 11-14 measurements/group. (**b**), (**c**), (**e**), and (**f**) *p* < 0.0001 (one-way analysis of variance); *p < 0.05, **p < 0.01, and ****p < 0.0001, TY11-15 control vs. 10D5 mAb treatment for matching *APOE* genotype (Sidak’s post hoc test). Differences between Age control and TY11-15 groups were non-significant for all matching *APOE* backgrounds (Sidak’s post hoc test); not shown on the graph. ^####^ *p* < 0.0001, TY11-15 APP/ε4 control vs. TY11-15 APP/ε2 or APP/ε3 controls (Sidak’s post hoc test). ^++^ *p* < 0.01 and ^++++^ *p* < 0.0001, 10D5 mAb treated APP/ε4 mice vs. 10D5 mAb treated APP/ε2 or APP/ε3 mice (Sidak’s post hoc test). ^♦^ *p* < 0.05, 10D5 mAb treated APP/ε2 mice vs. 10D5 mAb treated APP/ε3 mice (Sidak’s post hoc test). Scale bars 50 μm (**a**) and 25 μm (**d**)

Expression of two microglia markers, Iba1 and CD68 were compared across *APOE* genotypes and treatment arms. Iba1 is a calcium-binding protein specifically expressed by microglia and macrophages and its expression increases with microglia activation [30]. CD68 antigen is a glycoprotein localized to the lysosomal membrane in microglia and blood derived macrophages and is up regulated in actively phagocytic cells [31]. Activated microglia expressing both markers were found in the vicinity of Th-S positive plaques in control animals of all *APOE* genotypes but their loads were significantly higher in APP/ε4 mice compared to APP/ε2 and APP/ε3 mice. The load of Iba1 positive cells in the brain cortex of TY11-15 APP/ε4 control mice was 2.9 and 2.6 fold greater than in TY11-15 APP/ε2 and APP/ε3 control mice (*p* < 0.0001) (Fig. 3a, b), respectively; while the CD68 load was approximately 2.9 greater (*p* < 0.0001) (Fig. 3d, e). The isotype control antibody TY11-15 treatment had no significant effect on Iba1 and CD68 loads as their values were similar in TY11-15 control mice and untreated age-matched mice of corresponding *APOE* genotypes.

Passive immunization with 10D5 mAb effected significant microglia response across all *APOE* genotypes. The Iba1 load in 10D5 mAb treated APP/ε2, APP/ε3, and APP/ε4 mice was increased by 23.8% (*p* < 0.05), 39.0% (*p* < 0.05), and 39.4% (*p* < 0.0001) relative to the mean Iba1 load in TY11-15 controls of corresponding *APOE* genotypes, respectively (Fig. 3a, b); while the CD68 load was increased by 29.5% (*p* < 0.05), 32.8% (*p* < 0.05), and 23.3% (*p* < 0.0001), respectively (Fig. 3d, e).

Since the load of Iba1 and CD68 positive microglial was distinguishably the greatest in TY11-15 APP/ε4 mice, which also show the highest Aβ plaque load, we additionally analyzed the ratio of Iba1 and CD68 loads to the load of Th-S-positive parenchymal plaques for the same test area (Fig. 3c, f). This analysis enabled us to compare the level of microglia activation across *APOE* genotypes. We found no statistically significant differences in the Iba1/Th-S ratio and in the CD68/Th-S ratio between TY11-15 control and untreated age-matched control mice across all *APOE* genotypes suggesting that both Iba1 load and CD68 load correlate with fibrillar plaque load. 10D5 mAb immunization effected significant increase in both the Iba1/Th-S ratio and the CD68/Th-S ratio. The magnitude of increase was comparable between APP/ε2 and APP/ε3 genotypes while in APP/ε4 mice it was substantially higher. In 10D5 mAb treated APP/ε4 mice the Iba1/Th-S ratio became 1.4 fold higher than in 10D5 mAb treated APP/ε2 and APP/ε3 mice (*p* < 0.0001), while the CD68/Th-S ratio was 1.33 (*p* < 0.0001) and 1.21 (*p* < 0.01) fold higher, respectively (Fig. 3c, f).

Using the combination of anti-Iba1 immunostaining and Th-S counterstaining enabled us also to analyze morphology of microglia across *APOE* genotypes and treatment arms. Microglia associated with fibrillar plaques displaying features of activated microglia/macrophage ie. enlargement and rounding of the perikaryon and shortening of processes to their nearly disappearance, were observed in untreated age-matched and TY11-15 controls of all *APOE* backgrounds (Fig. 3a). 10D5 mAb treatment was associated with increased periplaque microglia recruitment and enhancement of its morphological activation features. This effect was significantly more pronounced in APP/ε4 mice than in other genotypes (Fig. 3a). In parallel to peri-plaque localization of activated microglia demonstrated by anti-Iba1 immunostaining, there also was noticeable peri-plaque localization of anti-CD68 immunostaining, which highlights lysosomal component of activated microglia/macrophages (Fig. 3d). Akin to Iba1 expression the peri-plaque expression of CD68 antigen increased with 10D5 mAb treatment across *APOE* genotypes but the increase was the most pronounced in 10D5 treated APP/ε4 mice. Peri-plaque recruitment of Iba1 positive activated microglia/macrophages and accompanying upsurge of CD68 expression in 10D5 mAb treated mice was associated with reduced plaque diameter as demonstrated by double anti-Iba1/Th-S and anti-CD68/Th-S stainings (Fig. 3a, d). We also observed Iba1 positive activated microglia and CD68 positive puncta, which were dissociated from Th-S positive plaque. They likely define activated microglia engaged with diffuse, immnunopositive Aβ plaques lacking fibrillar component. Their load also became markedly increased with 10D5 mAb treatment.

### *APOE* genotype has strong differential effect on VAβ burden and its reduction with Aβ immunotherapy

**Fig. 4:**
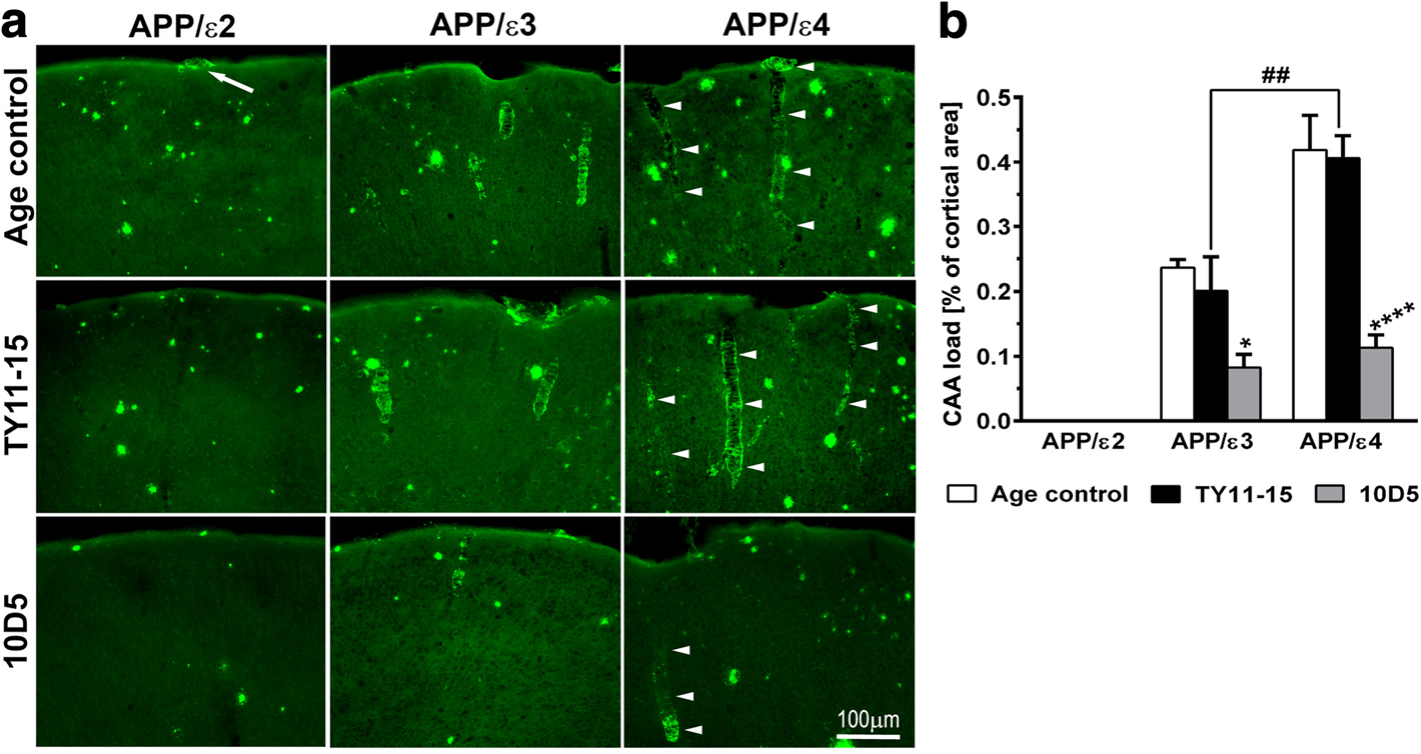
CAA analysis. *APOE* genotype differentially modulates the CAA load and its reduction during 10D5 mAb immunization. **a** Demonstration of CAA across *APOE* genotype spectrum. Representative microphotographs of Th-S stained coronal brain sections through the somatosensory cortex from untreated age-matched (Age control) mice, and mice receiving TY11-15 an isotype control IgG2a antibody or 10D5 anti-Aβ mAb. An arrow indicates a solitary CAA positive vessel in Age control APP/ε2 mouse. Arrowheads show long segments of CAA laden vessels in APP/ε4 mice and their reduction with 10D5 mAb treatment. **b** Mean (± SEM) of CAA load quantified in the brain cortex (*n* = 8-12/group). (**b**) *p* < 0.0001 (one-way analysis of variance); *p < 0.05, and ****p < 0.0001, TY11-15 control vs. 10D5 mAb treatment for matching *APOE* genotypes (Sidak’s post hoc test). ^##^ *p* < 0.01, APP/ε4 TY11-15 control vs. APP/ε3 TY11-15 control (Sidak’s post hoc test). Differences between Age control and TY11-15 groups were non-significant for matching *APOE* genotypes (Sidak’s post hoc test); not shown on the graph. Scale bar 100 μm (**a**)

**Fig. 5:**
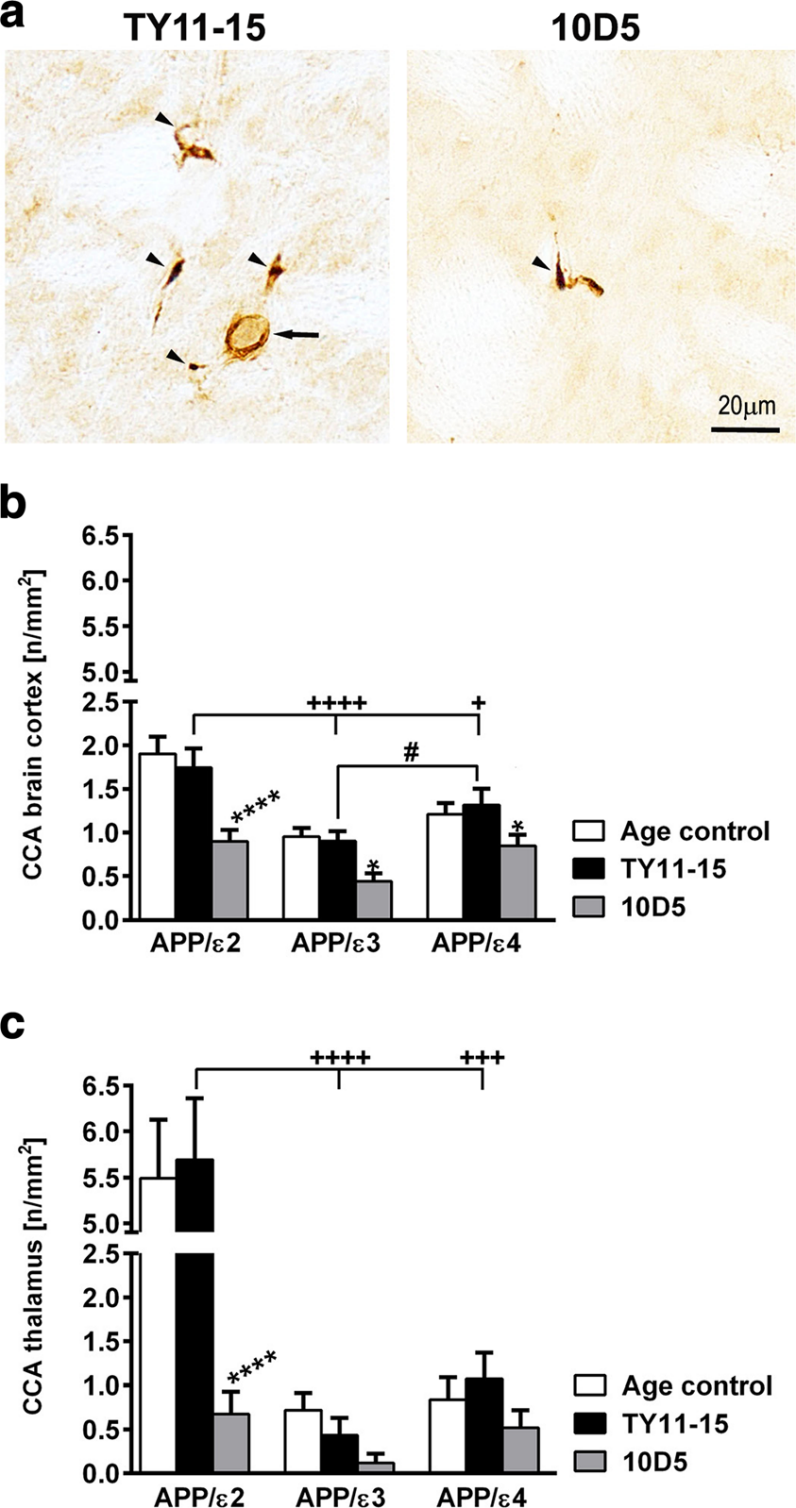
Characterization of CCA. The *APOE* ε2 allele is associated with increased CCA incidence. **a** Demonstration of CCA in the thalamus of APP/ε2 mice receiving TY11-15 isotype control IgG2a antibody or 10D5 anti-Aβ mAb. Arrowheads indicate numerous Aβ immunopositive capillary vessels (<8 μm in diameter). An arrow in the left panel points at the vessel, which wall is also Aβ immunopositive, but which was not counted as a capillary as its diameter exceeded the assumed capillary size cutoff. There is noticeable reduction in the density of Aβ immunoreactive capillaries following 10D5 mAb treatment shown on the right panel. Quantitative analysis of CCA. Shown are means (± SEM) for counts of Aβ immunopositive capillaries per mm^2^ in the brain cortex (**b**) and the thalamus (**c**) from 5–8 brains per *APOE* genotype and 3 sections per brain. *p* < 0.0001 (one-way analysis of variance) in (**b**) and *p* < 0.0001 (Kruskal-Wallis H test) in (**c**); *p < 0.05, and ****p < 0.0001, TY11-15 control vs. 10D5 mAb treatment for matching *APOE* genotypes (Sidak’s post hoc test in [**b**] and Dunn’s multiple comparison post hoc test in [**c**]). ^+^ *p* < 0.05, ^+++^ *p* < 0.001 and ^++++^ *p* < 0.0001, TY11-15 APP/ε2 control vs. TY11-15 APP/ε3 or TY11-15 APP/ε4 control groups (Sidak’s post hoc test in [**b**] and Dunn’s multiple comparison post hoc test in [**c**]). ^#^ *p* < 0.05 in (**b**), TY11-15 APP/ε3 control vs. TY11-15 APP/ε4 control (Sidak’s post hoc test). Differences between Age control and TY11-15 control for matching *APOE* genotypes in (**b**) and (**c**) were non-significant; not shown on the graph. Scale bar 20 μm (**a**)

Two forms of Aβ angiopathy were observed in brains of *APOE* targeted replacement APP_SWE_/PS1_dE9_ mice and they were analyzed separately. The first type was CAA defined as Th-S positive deposits of fibrillar Aβ in walls of meningeal arteries and arteries penetrating the brain cortex (Fig. 4a). CAA load was quantified in the brain cortex on Th-S stained sections and defined as the percent of the cross-sectional area of the cortex occupied by CAA laden vessels. The second type of Aβ angiopathy concerned immunoreactive Aβ deposits along walls of capillary vessels, which were Th-S negative (CCA) (Fig. 5a). CCA incidence was analyzed by calculating a number of Aβ immunopositive capillary profiles per mm^2^ in the brain cortex where they appeared in abundance across all *APOE* genotypes. In addition, CCA was analyzed in the thalamus as in APP/ε2 mice it was associated with outstanding number of perivascular hemosiderin deposits.

There was no significant difference in the CAA load between untreated age-matched and TY11-15 control mice for each of *APOE* genotypes analyzed. However across the TY11-15 control groups, APP/ε4 mice showed two fold higher CAA load than APP/ε3 mice (p < 0.01) (Fig. 4a, b). In contrast to APP/ε3 and APP/ε4 lines, in APP/ε2 mice CAA laden arteries were rarely occurring and were present in only some of the mice which precluded reliable quantification of the CAA load in this genotype (Fig. 4a). CAA laden vessels were rarely and inconsistently observed in the hippocampus and in subcortical structures including the thalamus in mice of any *APOE* genotype. 10D5 mAb treatment reduced the CAA load in the brain cortex in APP/ε3 and APP/ε4 mice by 56% (*p* < 0.05) and 71% (*p* < 0.01) relative to the mean CAA load value in TY11-15 controls of corresponding genotypes. However, given significantly higher CAA load baseline in APP/ε4 mice the absolute CAA load reduction in APP/ε4 mice was 2.5 fold higher than that in APP/ε3 mice. Thus, the CAA load in APP/ε4 animals was reduced from 0.41 ± 0.03% in TY11-15 controls to 0.11 ± 0.02% in 10D5 mAb treated group (absolute CAA load reduction = 0.30%) while in APP/ε3 mice the absolute reduction in the CAA load was 0.12%.

Unlike CAA, which was the most prevalent in APP/ε4 mice, the greatest abundance of CCA was associated with the *APOE* ε2 allele (Fig. 5a-c). The density of CCA associated capillary profiles per mm^2^ in the brain cortex of APP/ε2 TY11-15 control mice was 1.9 and 1.3 fold higher than these in the brain cortex of APP/ε3 (*p* < 0.0001) and APP/ε4 (*p* < 0.05) TY11-15 controls, respectively; while in the thalamus of APP/ε2 TY11-15 controls the average density of CCA associated capillary profiles per group was 13.1 and 5.3 fold higher than in the thalamus of APP/ε3 (*p* < 0.0001) and APP/ε4 (*p* < 0.001) TY11-15 control mice, respectively. There was no statistically significant difference in the CCA incidence between untreated age-matched and TY11-15 control mice in the brain cortex and in the thalamus for any of matching *APOE* genotype. One needs to mention that CCA was absent in the thalamus of two thirds of control APP/ε3 mice and of one half of control APP/ε4 mice. Besides the thalamus, the incidence of CCA in other subcortical structures was scanty especially in APP/ε3 and APP/ε4 mice, thus no other structure specific quantification was performed.

10D5 mAb treatment effected significant CCA reduction across all three *APOE* genotypes. In the brain cortex of APP/ε2 and APP/ε3 mice the density of Aβ immunopositive capillary profiles was reduced approximately two-fold (*p* < 0.001 and *p* < 0.05; respectively), while in the brain cortex of APP/ε4 mice it was reduced by 1.6 fold (*p* < 0.05). 10D5 mAb treatment reduced CCA in the thalamus of APP/ε2 mice 8.5 fold (*p* < 0.0001). Marked reduction also was observed in the thalamus of APP/ε3 and APP/ε4 mice, but it did not reach statistical significance as the data did not conform to normal distribution hence weaker non-parametric statistical analysis was used.

### *APOE* ε2 and ε3 alleles show opposing effect on the risk of cerebral microhemorrhages

**Fig. 6:**
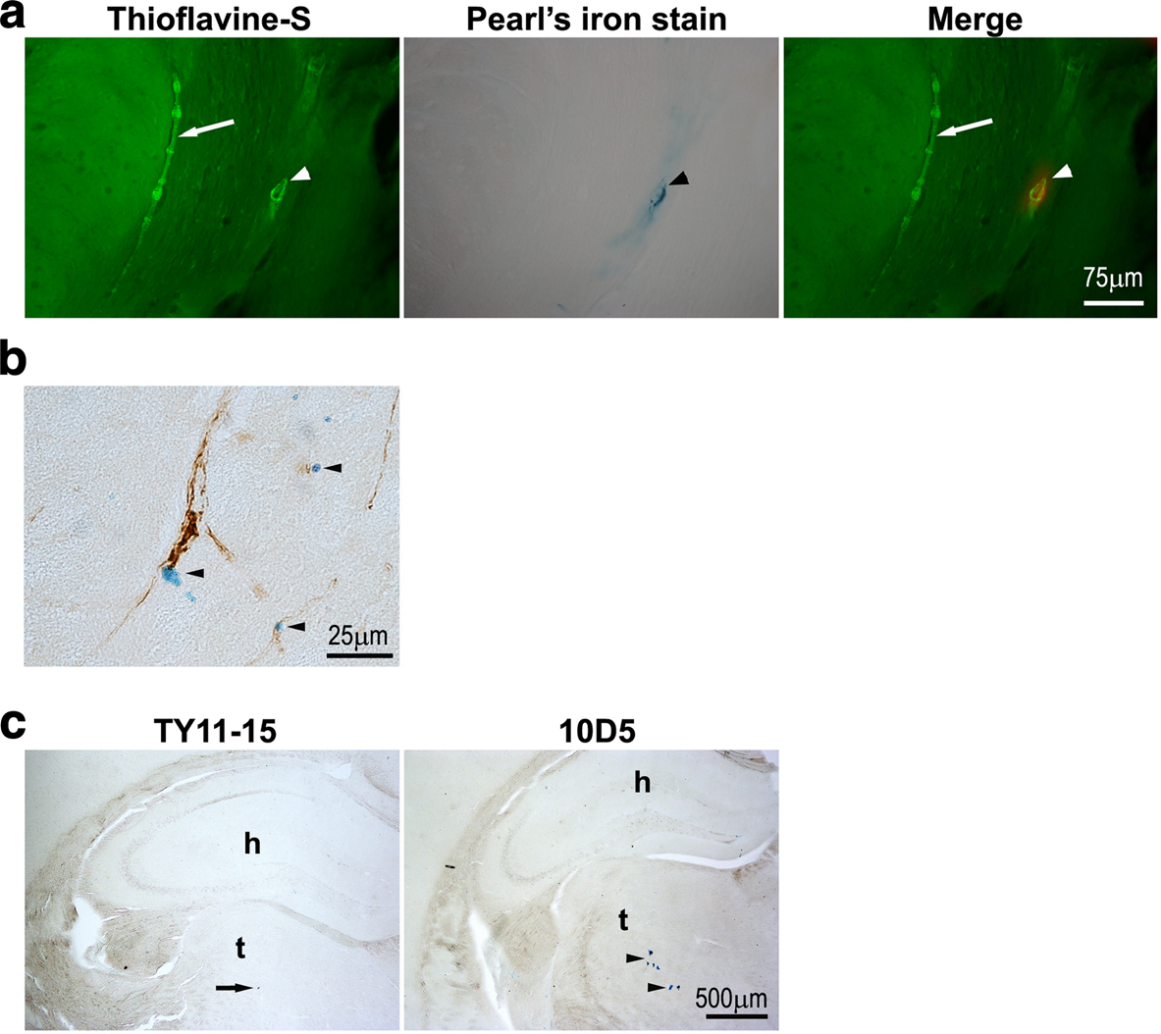
Demonstration of perivascular hemosiderin deposits reflecting ensued microhemorrhages. **a** Shown is an example of hemosiderin deposition associated with CAA laden vessel in the cerebral cortex of 10D5 mAb treated APP/ε4 animal. The histological section was double-stained with Thioflavin-S and Pearls’ Prussian blue stain for ferric iron and photographed under fluorescence and bright-field microscopy, respectively. Positive perivascular hemosiderin stain (arrowhead) was digitally converted to red color and superimposed on green Thioflavin-S stain of CAA (Merge). An arrow indicates a vessel affected by CAA, without evidence of bleeding. **b** Representative microphotograph of CCA related hemosiderin deposits (arrowheads) in the brain cortex of 10D5 mAb-treated APP/ε2 mouse. A coronal section of the cerebral cortex was immunostained with anti-Aβ HJ3.4 mAb and counterstained with Pearls’ Prussian blue stain. **c** Examples of large hemosiderin deposits in the thalamus typical of APP/ε2 mice; Pearl’s iron stain without counterstain. An arrow on the left panel indicates an isolated thalamic hemosiderin deposit in TY11-15 control APP/ε2 animal, while arrowheads on the right panel point at the cluster of large hemosiderin deposits (≥15 μm in diameter) identified in 10D5 mAb treated APP/ε2 mouse. Abbreviations: h-hippocampus, t-thalamus. Scale bars: 75 μm (**a**), 25 μm (**b**), 500 μm (**c**)

**Fig. 7:**
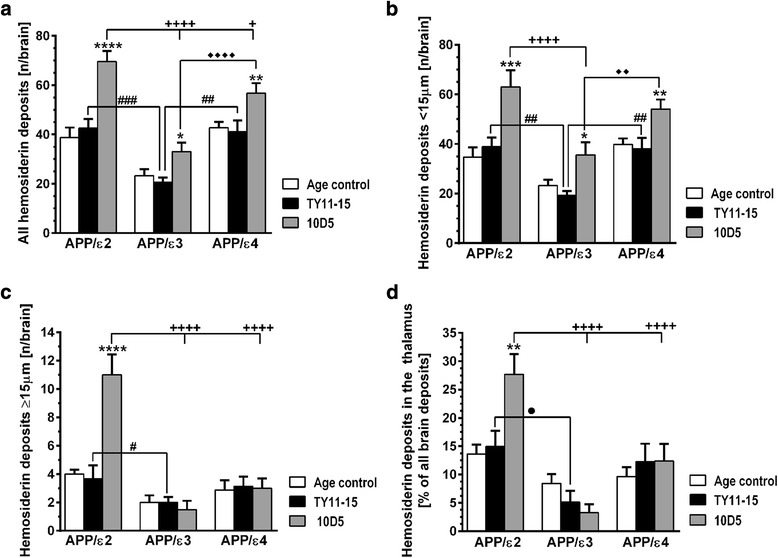
Quantitative analysis of perivascular hemosiderin deposits. *APOE* genotype differentially modulates occurrence of hemosiderin deposits and their increase during Aβ immunotherapy with *APOE* ε2 and *APOE* ε3 alleles having adverse and protective effects, respectively. Shown are means (± SEM) for counts of all brain perivascular hemosiderin deposits (**a**), small perivascular hemosiderin deposits (<15 μm in diameter) (**b**), large perivascular hemosiderin deposits (≥15 μm in diameter) (**c**), and perivascular hemosiderin deposits in the thalamus presented as per cent of all brain deposits (**d**) (n = 5-11/group). Hemosiderin deposits were counted on every tenth brain coronal cross-section along the entire rostro-caudal axis of the brain. (**a**) through (**d**) *p* < 0.0001 (one-way analysis of variance); *p < 0.05, **p < 0.01, ***p < 0.001, and ****p < 0.0001, TY11-15 control vs. 10D5 mAb treatment for matching *APOE* genotypes (Sidak’s post hoc test). ^+^ *p* < 0.05, and ^++++^ *p* < 0.0001, 10D5 mAb treated APP/ε2 mice vs. other 10D5 mAb treated APP/ε3 or APP/ε4 mice (Sidak’s post hoc test). ^♦♦^ *p* < 0.01, and ^♦♦♦♦^ *p* < 0.0001, 10D5 mAb treated APP/ε3 mice vs. 10D5 treated APP/ε4 mice (Sidak’s post hoc test). ^●^ *p* < 0.05 APP/ε2 TY11-15 control vs. APP/ε3 TY11-15 control (Sidak’s post hoc test). Differences between Age control and TY11-15 groups were non-significant for all types of hemosiderin deposits for matching *APOE* backgrounds, so were differences in the count of large hemosiderin deposits between TY11-15 control and 10D5 mAb treated APP/ε3 and APP/ε4 mice (Sidak’s post hoc test); not shown on the graph

Perivascular hemosiderin deposits, reflecting ensued microhemorrhages, were found associated both with CAA and CCA positive vessels (Fig. 6a, b). They frequently occurred in untreated age-matched mice of all *APOE* genotypes and their number did not increase in TY11-15 treated control animals (Fig. 7a-d). There were no statistically significant differences in the number of the total brain hemosiderin deposits, the number of deposits subclassified as small and large (with cut off ≥ 15 μm in diameter), and the fraction of deposits localized to the thalamus between TY11-15 treated APP/ε2 and APP/ε4 mice (Fig. 7a-d). In contrast, untreated age-matched and TY11-15 APP/ε3 control mice had approximately two-fold less hemosiderin deposits for each listed category compared to APP/ε2 and APP/ε4 TY11-15 controls.

10D5 mAb treatment resulted in a significant increase in the number of hemosiderin deposits yet the magnitude of this effect was differentially modulated by the *APOE* genotype. In APP/ε3, and APP/ε4 mice, an increase by 12.4 and 15.6 deposits per brain on average was observed, respectively; bringing their number in APP/ε3 mice from 20.5 ± 1.9 per brain in TY11-15 controls to 32.9 ± 3.8 per brain in 10D5 mAb treatment group (*p* < 0.05), while in APP/ε4 mice, from 41.1 ± 4.6 per brain in TY11-15 controls to 56.7 ± 4.1 per brain in the 10D5 mAb treatment group (*p* < 0.01) (Fig. 7a). The increase only concerned small deposits (Fig. 7b), while the number of large deposits did not change in either APP/ε3 or APP/ε4 mice (Fig. 7b, c). Differences in the number of all hemosiderin deposits and small hemosiderin deposits between 10D5 mAb treated APP/ε3 and APP/ε4 mice were statistically significant (*p* < 0.0001 and *p* < 0.01, respectively) (Fig. 7a, b). In APP/ε2 mice 10D5 mAb treatment caused a nearly two-fold higher upsurge in the number of hemosiderin deposits compared to APP/ε3 and APP/ε4 mice. The count of all hemosiderin deposits was increased on averaged by 27.0 per brain bringing it from 42.5 ± 3.8 per brain in TY11-15 controls to 69.5 ± 4.4 per brain in 10D5 mAb treated group (*p* < 0.0001) (Fig 7a, b). Unlike other *APOE* genotypes, APP/ε2 mice experienced an increase both in the number of small and large hemosiderin deposits. The count of large hemosiderin deposits was increased on average by 7.3 per brain from 3.7 ± 0.9 in TY11-15 controls to 11.0 ± 1.4 in 10D5 treated mice (a nearly three-fold increase) (*p* < 0.0001) (Fig 7c).

The bulk of perivascular hemosiderin deposits was found in the brain cortex (Additional file 2: Figure S2 A-C). In Age control and TY11-15 control APP/ε3 and APP/ε4 mice they constituted between 65 to 70% of the brain total hemosiderin deposits, while in Age control and TY11-15 control APP/ε2 mice 53% and 58%, respectively. As compared to other *APOE* genotypes APP/ε2 mice showed greater number of hemosiderin deposits to the thalamus. In APP/ε2 TY11-15 control mice 14.9 ± 2.8% of all brain hemosiderin deposits was found in the thalamus compared to 5.1 ± 1.9% in TY11-15 APP/ε3 controls (*p* < 0.05) and 12.3 ± 3.1% in TY11-15 APP/ε4 controls (non-significant) (Fig. 7d). While in APP/ε3 and APP/ε4 mice 10D5 mAb treatment did not affect the proportion of thalamic hemosiderin deposits to all brain deposits, in 10D5 mAb treated APP/ε2 mice this proportion nearly doubled and it was 27.7 ± 3.6% on average (*p* < 0.0001) (Fig. 7d; Additional file 2: Figure S2 A). Except for minor increase in the number of hemosiderin deposits in the hippocampus in APP/ε2 mice, no other brain structure but the brain cortex in animals of all *APOE* genotypes and the thalamus in APP/ε2 mice only, showed significant regional increase in the number of hemosiderin deposits with anti-Aβ passive immunization (Additional file 2: Figure S2 A-C).

### *APOE* alleles have differential effect on cerebrovascular disease risk factors in APP_SWE_/PS1_dE9_ mice

**Fig. 8:**
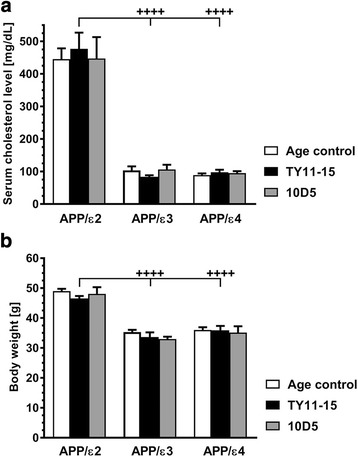
Analysis of cerebrovascular disease risk factors. APP/ε2 mice show hypercholesterolemia and obesity. Mean ± SEM of the total serum cholesterol level (**a**) (*n* = 5-10/group) and body weight (**b**) (*n* = 6-16/group) of untreated Age control, TY11-15 treated control, and 10D5 mAb treated mice determined at the age of 15 months at the conclusion of the experiment. **a** and (***b***) *p* < 0.0001 (one-way analysis of variance); ^++++^ *p* < 0.0001, TY11-15 APP/ε2 vs. TY11-15 APP/ε3 and APP/ε4 control mice (Sidak’s post hoc test). Differences between Age control, TY11-15 control, and 10D5 mAb treated mice were non-significant for matching *APOE* backgrounds (Sidak’s post hoc test); not shown on the graph

*APOE* ε2 targeted replacement mice are established model of type III hyperlipidemia. APP/ε2 TY11-15 control mice used in this study showed average total serum cholesterol level of 477.3 ± 49.5 mg/dl at the age of 15 months. For comparison total serum cholesterol level in TY11-15 control APP/ε3 and APP/ε4 mice was 83.8 ± 4.7 mg/dL and 97.9 ± 7.7 mg/dL, respectively (*p* < 0.0001 vs. APP/ε2) (Fig. 8a ). APP/ε2 mice were also obese. The average weight of APP/ε2 TY11-15 control mice was 46.6 ± 0.8 g, while average weight of TY11-15 control APP/ε3 and APP/ε4 mice was 33.6 ± 1.6 g and 35.9 ± 1.5 g, respectively (*p* < 0.0001 vs. APP/ε2) (Fig. 8b). Neither serum cholesterol level nor animal weight was significantly changed with 10D5 mAb treatment. No significant differences in respect to total serum cholesterol level and animal body weight between untreated age-control and TY11-15 control mice for matching *APOE* genotypes were observed.

## Discussion

*APOE* genotype critically affects the burden of Aβ pathology in sporadic AD. ApoE isoforms encoded by various *APOE* alleles differentially influence the rate of soluble Aβ clearance from the brain interstitial space and formation of Aβ parenchymal plaques and VAβ [32, 33]. Our study provides novel evidence that *APOE* genotype also differentially affects multiple aspects of response to Aβ immunotherapy. Firstly, we observed that *APOE* genotype is associated with variable reduction in the load of Aβ parenchymal plaques and APP/ε4 mice had greater reduction in absolute Aβ plaque load values than APP/ε2 and APP/ε3 mice. The reduction concerned both Th-S positive, (fibrillar) and immunopositive Aβ (total) plaque loads with the later metric showing greater change than the former across all *APOE* genotypes. As the immunopositive Aβ plaque load is inclusive of Th-S positive plaques this suggests that stimulated by 10D5 mAb microglia clear diffuse component of Aβ plaques more effectively than they do fibrillar Aβ aggregates. However, despite enhanced treatment effect in APP/ε4 mice the post-treatment Aβ plaque load in these mice and especially the fibrillar Aβ plaque load, remained significantly higher than in APP/ε2 and APP/ε3 mice, what is a result of distinguishably greater Aβ plaque load, associated with the ε4 allele. This observation may inform design of future passive immunization experiments in APP Tg model mice of AD and clinical trials suggesting specific tailoring of the anti-Aβ passive immunization protocol in *APOE* ε4 allele carriers to achieve end-point plaque load reduction level compared to that expected among non-ε4 allele carriers. Potential modifications of the immunization protocol may include increasing antibody dose, earlier commencement of the treatment and extension of treatment duration. However one needs to be mindful that escalation of anti-Aβ mAbs dose in *APOE* ε4 allele carriers may trigger increased rate of vasculotropic adverse events associated with immunization [5, 8, 14]. With the advancement of certain newer anti-Aβ immunotherapies such as Gantanerumab and Aducanumab that appear to lower Aβ plaque load in humans to a greater extent than seen previously [6, 8], it will be interesting to see if the effects of *APOE* genotype noted here in animal models are also present in humans.

The primary mechanism of action of 10D5 mAb is based on its direct binding to deposited Aβ and stimulating Fc receptor-mediated Aβ plaque removal by activated microglia and possibly blood derived macrophages [9, 34]. To compare microglial response across *APOE* genotypes we immunostained brain sections against Iba1 and CD68 antigens and counterstained them with Th-S for fibrillar Aβ. Iba1 is a microglia specific marker, which expression increases with microglia activation and macrophage transformation [30], while CD68 is a lysosomal/endosomal membrane glycoprotein up regulated in actively phagocytic cells [31]. The load of Iba1 and CD68 positive cells was significantly higher in Age control and TY11-15 control APP/ε4 mice compared to APP/ε2 and APP/ε3 controls. Both Iba1 and CD68 loads correlated with the load of Th-S positive fibrillar deposits and when adjusted for it, they were comparable across all *APOE* genotypes in control animals. Following 10D5 mAb treatment both Iba1 and CD68 loads increased across all *APOE* genotypes, however when adjusted for the post-treatment fibrillar plaque load, they were significantly higher in APP/ε4 mice than in APP/ε2 and APP/ε3 animals. This finding illustrates increased effector function of 10D5 mAb exerted on microglia in the setting of the *APOE* ε4 allele. In particular exaggerated post treatment CD68/Th-S ratio in APP/ε4 mice implies less effective Aβ degradation, which can be tied to a recent discovery showing that excessive apoE accumulation within Aβ plaques impairs ability of microglia to contain plaque load [35]. In fact, we have previously demonstrated that APP/ε4 and PDAPP/ε4 mice have greatly increased content of apoE within Aβ deposits compared to the same Tg mice lines expressing other human *APOE* alleles [19, 36]. Though microglial cells play pivotal role in maintaining immunoproteostasis through phagocytosis and degradation of misfolded proteins, their protective function is inseparably associated with immune activation exerting a chronic, potentially harmful effect on the regional milieu of neuronal networks [35, 37–39]. Although, APP/ε4 mice show the greatest absolute reduction in the load of parenchymal Aβ plaques in response to anti-Aβ immunization as compared to mice of other *APOE* genotypes, this occurs at the expense of unparalleled microglia response making the post treatment Iba1 and CD68 loads the highest among all experimental groups. Thus both higher level of microglia activation driven by higher Aβ plaque load and exaggerated microglia effector response exerted by anti-Aβ immunotherapy may constitute independent deleterious effects associated with the ε4 allele, in AD pathogenesis. Increased microglia activation and associated reduction in Aβ plaque load was found only in 10D5 mAb immunized groups, while administration of the isotype control antibody TY11-15 changed neither expression of microglia markers nor parenchymal plaque load as compared to untreated age-matched animals of matching *APOE* genotypes.

Both CAA and CCA variants of VAβ pathology have been described in AD autopsies and their relative preponderance has been linked to various *APOE* genotypes [40–43]. CAA comprises of fibrillar Aβ sandwiched between the adventitia and the tunica media of cerebral arteries, while CCA comprises of non-fibrillar Aβ deposits closely associated with the capillary outer basement membrane. Both development of CAA and CCA is primarily driven by neuron derived Aβ, while Aβ produced by myocytes locally in the tunica media further contributes to CAA development (reviewed in [42]). Presence of apoE within CAA lesions is critical for formation of Aβ fibrillar assemblies [25]. In this study we found that *APOE* genotype modulates development of both CAA and CCA pathology in APP Tg mice. In accordance to previously published data concerning ε4 allele effect, we found increased CAA load in APP/ε4 mice [25, 44–46]. Our novel observation here is that the ε2 allele was associated with greatly reduced CAA load, but also with enhanced incidence of CCA. Reduced CAA load in APP/ε2 mice likely reflects limited effect of apoE2 isoform on promoting Aβ fibrillar assemblies within arterial walls as compared to other human apoE isoforms, which are associated with higher load of CAA and fibrillar Aβ plaques [47]. In turn, greater incidence of CCA may be related to increased amount of soluble Aβ cleared across the BBB in APP/ε2 animals, which deposit less Aβ in the brain while maintaining the same level of Aβ production [32]. In fact increased rate of soluble Aβ clearance across the BBB has been experimentally confirmed in the setting of the *APOE* ε2 allele [32, 48]. Vascular disease risk factors including hypercholesterolemia and volume overload occurring in aged APP/ε2 mice effect deterioration of capillary wall structure and function and likely in tandem with greater load of soluble Aβ passing across the BBB contribute to CCA development.

The largest percentage of AD patients with both CAA and CCA was found among ε4 allele carriers, while CAA without CCA was more frequent among ε2 and ε3 carriers [40, 42]. In these studies ε3 allele carriers and in particular ε3 allele homozygotes constituted the largest percentage among CAA/CCA free cases. Accordingly, we found that APP/ε3 mice have the lowest incidence of CCA among all *APOE* genotypes and their CAA load is significantly lower than that in APP/ε4 mice demonstrating protective effect of the ε3 allele against development of Aβ angiopathy. However, mechanisms underlying VAβ deposition including preferential development of CAA and CCA remain elusive and require further elucidation. They likely are a resultant of multiple genetic features extending beyond *APOE* genotype as no *APOE* allelic variant confers absolute protection against VAβ deposition [42, 43].

VAβ is detrimental to the function of cerebrovascular circulation through compromising blood–brain-barrier integrity [49], effecting hemodynamic dysfunction [29, 50], and facilitating occurrence of spontaneous hemorrhages [25, 26, 36]. We found a significant number of perivascular hemosiderin deposits, reflecting ensued microhemorrhages, in brains of control mice representing all *APOE* genotypes. They were associated both with CAA and CCA affected vessels. Strikingly the lowest incidence of perivascular hemosiderin deposits was observed among untreated age-matched and TY11-15 control APP/ε3 mice. In APP/ε4 and APP/ε2 controls the incidence of perivascular hemosiderin deposits was comparable and statistically significant higher than that in APP/ε3 controls. In human subjects prevalence of spontaneous microhemorrhages is high and positively correlates with advancing age. MRI studies detected microbleeds in 17.8% of subjects aged 60–69 years and in 38.3% in subjects who were more than 80 years old [51, 52]. For comparison an autopsy study reported histological evidence of ensued microhemorrhages in 92.9% of elderly individuals with mean age of 81.1 ± 10.8 years, which included both AD and non-demented subjects [53]. Evidence independently supports link of both the *APOE* ε4 and ε2 allele with increased risk for microhemorrhages in humans [51, 52, 54–57]. Both VAβ and hypertensive or atherosclerotic microangiopathy are recognized clinical risk factors for cerebral microbleeds, with the former correlating stronger with bleeding in cortical/subcortical distribution [58, 59] while the later with bleeding to deep brain structures including basal ganglia and thalamic nuclei [60, 61]. *APOE* ε2 targeted replacement mice develop type III hyperlipoproteinemia and spontaneous atherosclerosis [16] as well as volume overload as a function of their obesity, all of which are recognized vascular risk factors and may explain increased preponderance to thalamic localization of hemosiderin deposits in APP/ε2 mice found in our study. However, these striking vasculotropic effects observed here in APP/ε2 mice may not be robustly seen in human subjects, where ε2 homozygotes constitute only 1% of the population, with less than 10% of them developing type III hyperlipoproteinemia, what is linked to distinctly different contribution of apoE to VLDL formation between humans and mice [62].

Aβ immunotherapy can ameliorate VAβ burden and improve compromised vascular reactivity [12, 21, 63], but it is well known to escalate incidence of brain microhemorrhages [21, 26]. Both CAA load and CCA incidence were reduced in 10D5 mAb treated mice with the strongest effect on CAA load reduction seen in APPε4 animals and the strongest effect on CCA incidence seen in APP/ε2 mice. We also found a profound effect of *APOE* genotype on Aβ immunization related microhemorrhages. Surprisingly not the ε4 allele but the ε2 allele was associated with the greatest increase in the number of perivascular hemosiderin deposits. 10D5 mAb treated APP/ε2 mice had the highest increase in the number of all perivascular hemosiderin deposits and also in the number of deposits in small and large (≥15 μm in diameter) subcategories. The increase in the total number of hemosiderin deposits in APP/ε2 mice was approximately two-fold higher than these in APP/ε3 and APP/ε4 mice. APP/ε2 mice represented the only *APOE* genotype where 10D5 mAb immunization increased incidence of large hemosiderin deposits, bringing their number to three-fold value of the control group. Several human autopsy studies have shown that the ε2 allele is an exacerbating factor of microhemorrhages among subjects with VAβ pathology and that this effect is linked to fibrinoid necrosis of vascular wall specifically associated with the ε2 allele [55–57]. As mentioned above APP/ε2 mice also develop hypercholesterolemia and atherosclerosis, hence increased susceptibility to cerebral microhemorrhages and in particular high incidence of large hemosiderin deposits in APP/ε2 mice associated with immunotherapy can be a resultant of prevalent VAβ pathology, anti-Aβ mAb effect, and cerebrovascular risk factors all cooperatively compromising vascular wall integrity. In contrast, 10D5 mAb treated APP/ε3 and APP/ε4 mice showed only an increase in the number of small hemosiderin deposits. Again the post-10D5 treatment number of hemosiderin deposits in APP/ε3 mice was the lowest among all *APOE* genotypes, indicating a relative vasculoprotective effect of the ε3 allele, which has not been previously described in AD patients or APP Tg mice expressing human *APOE* alleles.

Exacerbation of VAβ associated microhemorrhages appears to be associated with propensity of anti-Aβ mAb to bind deposited Aβ. Thus, application of N-terminus specific mAbs, which like10D5 binds epitopes exposed in Aβ parenchymal plaques and VAβ, but not mAbs, which like m266 bind the Aβ central domain and do not bind to deposited Aβ effect increased hemorrhage incidence [64, 65]. However mAbs binding deposited Aβ are generally more effective in clearing VAβ [12]. Time course analysis of VAβ-related events during immunotherapy in PDAPP mice, revealed that a time window for increased incidence of microhemorrhages can last up to six months, but then their incidence diminishes drastically, which correlates with achieving significant clearance of both CAA and CCA [21]. Thus, the risk of brain microhemorrhages related to Aβ immunotherapy may be a transient complication and possibly reduced in individuals with modest VAβ burden, who also have lower prevalence of spontaneous microbleeds [58, 59].

## Conclusions

Utilizing *APOE* humanized APP_SWE_/PS1_dE9_ mice we demonstrated that *APOE* alleles differentially modulate various aspects of response to anti-Aβ immunotherapy including Aβ load reduction, microglia response, and incidence of cerebral microhemorrhages. This information can be utilized in design of clinical trials of anti-Aβ mAbs and other Aβ targeting therapeutics in order to maximize potency of studied approaches and minimize adverse reaction to the treatment.

## Additional files

Additional file 1: Figure S1. Comparative analysis of Aβ plaque load immunostained against N-terminal and central Aβ epitopes revealed no significant differences. **a** Representative microphotographs of coronal brain sections through the somatosensory cortex from the same TY11-15 control, and 10D5 mAb treated mice of APP/ε4 background, which were immunostained using HJ3.4 mAb directed against the N-terminus of Aβ and 4G8 mAb directed against the mid-portion of Aβ. **b** Unbiased analysis of the parenchymal Aβ plaque load in the brain cortex revealed by HJ3.4 and 4G8 immunostaining. Values represent mean ± SEM from 10 to 12 animals per group. **b** *p* < 0.0001 (one-way analysis of variance); *****p* < 0.0001, TY11-15 control vs. 10D5 mAb treatment for matching anti-Aβ immunostains (Sidak’s post hoc test). Differences between HJ3.4 and 4G8 immunostaining in TY11-15 control and 10D5 mAb treatment groups were non-significant; not shown on the graph. Scale bar 50 μm (**a**). (PDF 423 kb)
Additional file 2: Figure S2. Regional analysis of perivascular hemosiderin deposits identifies the brain cortex in mice of all *APOE* genotypes and the thalamus in APP/ε2 mice as brain structures susceptible to microbleeds associated with anti-Aβ immunization. Shown are means (± SEM) for counts of all brain perivascular hemosiderin deposits in the brain cortex, the hippocampus, the basal ganglia, the corpus callosum, the thalamus, the septum and the midbrain in APP/ε2 (**a**), APP/ε3 (**b**), and APP/ε4 mice (**c**) (n = 5-11/group). Perivascular hemosiderin deposits were counted on every tenth brain coronal cross-section along the entire rostro-caudal axis of the brain. **a** through (**c**) *p* < 0.0001 (one-way analysis of variance); **p* < 0.05, ****p* < 0.001, and *****p* < 0.0001, TY11-15 control vs. 10D5 mAb treatment for matching brain structures and *APOE* genotypes (Sidak’s post hoc test). For brain structures where the differences between TY11-15 control and 10D5 mAb treatment groups were not statistically significant *p* values are not shown. Differences between Age control and TY11-15 groups were non-significant for all structures (Sidak’s post hoc test); *p* values not shown on the graph. (PDF 419 kb)

## Abbreviations

AD: Alzheimer’s disease
*APOE*: human apolipoprotein E gene
*Apoe*: murine apolipoprotein E gene
apoE: apolipoprotein E
APP: APP_SW_/PS1_dE9_ transgene
ARIA: amyloid related imaging abnormalities
Aβ: β-amyloid
BBB: blood–brain barrier
CAA: cerebral amyloid angiopathy
CCA: cerebral capillary Aβ
DAB: 3,3’-diaminobenzidine
FA: formic acid
h: hippocampus
mAb: monoclonal antibody
PBS: phosphate buffered saline
PET: positron emission tomography
SEM: standard error of the mean
t: thalamus
Tg: transgenic
Th-S: Thioflavine-S
VAβ: vascular Aβ
