# Supplementary figures and images for: APOE Genotype Differentially Modulates Effects of Anti-Aβ, Passive Immunization in APP Transgenic Mice
Source: Mol Neurodegener. 2017 Jan 31;12:12. doi: 10.1186/s13024-017-0156-1 (PMC5282859; doi:10.1186/s13024-017-0156-1)

**Figure S1**

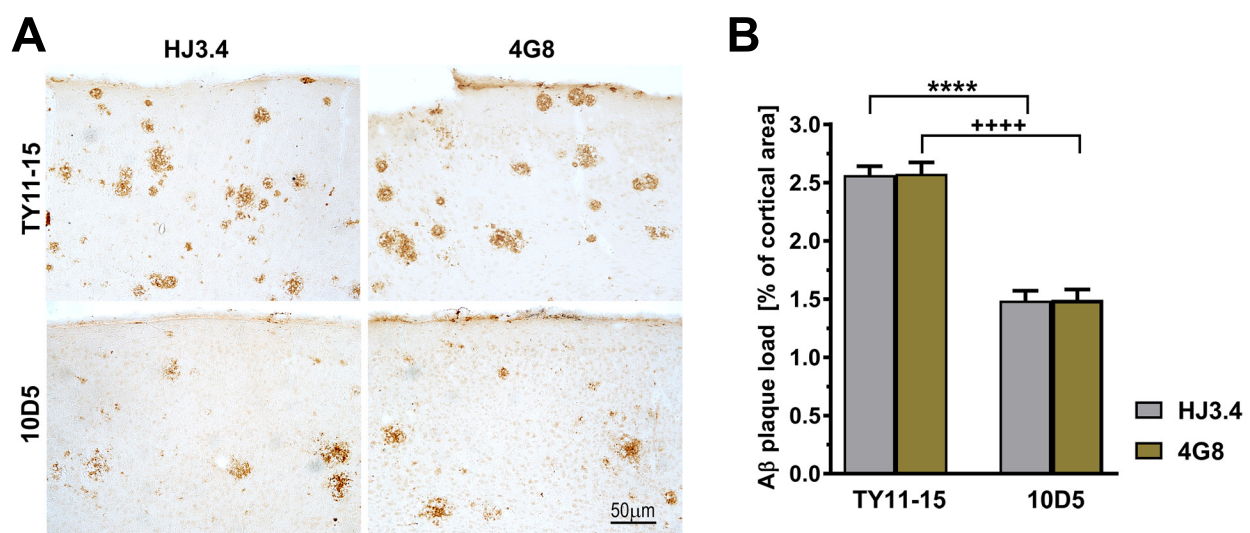

Supplement: Additional file 1: Figure S1. — Comparative analysis of Aβ plaque load immunostained against N-terminal and central Aβ epitopes revealed no significant differences. a Representative microphotographs of coronal brain sections through the somatosensory cortex from the same TY11-15 control, and 10D5 mAb treated mice of APP/ε4 background, which were immunostained using HJ3.4 mAb directed against the N-terminus of Aβ and 4G8 mAb directed against the mid-portion of Aβ. b Unbiased analysis of the parenchymal Aβ plaque load in the brain cortex revealed by HJ3.4 and 4G8 immunostaining. Values represent mean ± SEM from 10 to 12 animals per group. b p < 0.0001 (one-way analysis of variance); ****p < 0.0001, TY11-15 control vs. 10D5 mAb treatment for matching anti-Aβ immunostains (Sidak’s post hoc test). Differences between HJ3.4 and 4G8 immunostaining in TY11-15 control and 10D5 mAb treatment groups were non-significant; not shown on the graph. Scale bar 50 μm (a). (PDF 423 kb) [file 13024_2017_156_MOESM1_ESM.pdf]

# Figure S2

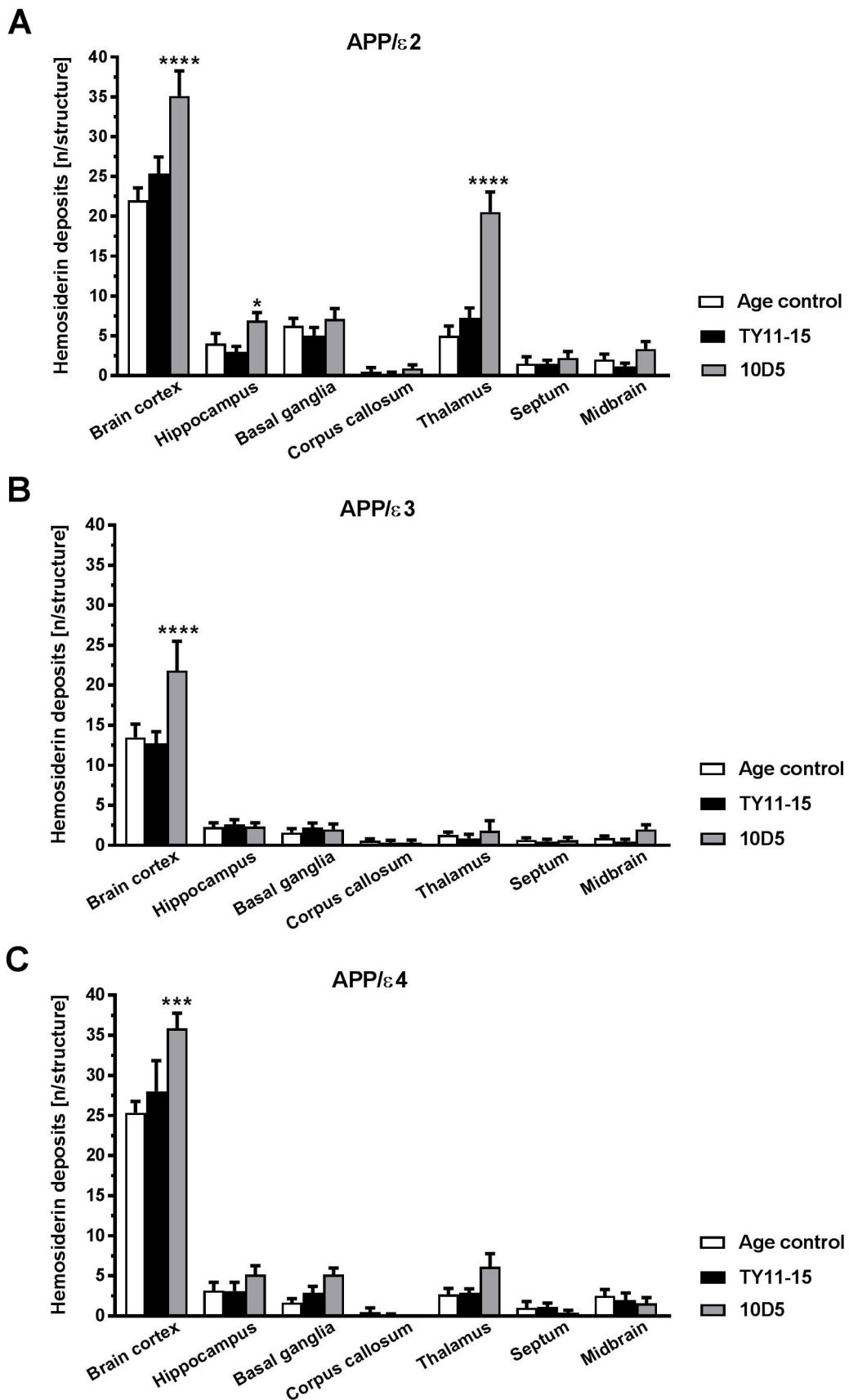

Supplement: Additional file 2: Figure S2. — Regional analysis of perivascular hemosiderin deposits identifies the brain cortex in mice of all APOE genotypes and the thalamus in APP/ε2 mice as brain structures susceptible to microbleeds associated with anti-Aβ immunization. Shown are means (± SEM) for counts of all brain perivascular hemosiderin deposits in the brain cortex, the hippocampus, the basal ganglia, the corpus callosum, the thalamus, the septum and the midbrain in APP/ε2 (a), APP/ε3 (b), and APP/ε4 mice (c) (n = 5-11/group). Perivascular hemosiderin deposits were counted on every tenth brain coronal cross-section along the entire rostro-caudal axis of the brain. a through (c) p < 0.0001 (one-way analysis of variance); *p < 0.05, ***p < 0.001, and ****p < 0.0001, TY11-15 control vs. 10D5 mAb treatment for matching brain structures and APOE genotypes (Sidak’s post hoc test). For brain structures where the differences between TY11-15 control and 10D5 mAb treatment groups were not statistically significant p values are not shown. Differences between Age control and TY11-15 groups were non-significant for all structures (Sidak’s post hoc test); p values not shown on the graph. (PDF 419 kb) [file 13024_2017_156_MOESM2_ESM.pdf]
